# Effects of Diabetes on Inflammatory Status and Prognosis in Cancer Patients

**DOI:** 10.3389/fnut.2022.792577

**Published:** 2022-03-04

**Authors:** Xiangliang Liu, Kaiwen Zheng, Wei Ji, Wenxin Zhang, Yuguang Li, Mingyang Liu, Jiuwei Cui, Wei Li

**Affiliations:** ^1^Cancer Center, The First Hospital of Jilin University, Changchun, China; ^2^Department of Radiation and Medical Oncology, Zhongnan Hospital, Wuhan University, Wuhan, China; ^3^College of Instrumentation and Electrical Engineering, Jilin University, Changchun, China

**Keywords:** cancer, diabetes, inflammation, prognosis, BMI

## Abstract

**Background:**

Cancer and diabetes mellitus (DM) are prevalent, but there still a lack of convinced evidence clearly explaining the extent of the effect of diabetes in cancer.

**Data and Methods:**

Clinical data of 2,929 cancer patients were collected. Diabetes were diagnosed according to the Diabetes Diagnosis and Treatment Criteria. BMI was classified by the BMI standards for Chinese adults published by the Working Group on Obesity. All involved patients were classified into the non-DM group and DM group. The Kaplan–Meier curve, log-rank test and Cox regression analyses were used to perform survival analysis.

**Results:**

Compared with non-DM patients, OS in DM patients was significant shorter in lung cancer (HR = 2.076, *P* = 0.001 in early stage; HR = 2.118, *P* < 0.001 in advanced stage), digestive tract cancer (HR = 1.768, *P* = 0.020 in early stage; HR = 2.454, *P* = 0.005 in advanced stage), leukemia (HR = 2.636, *P* < 0.001), breast cancer (HR = 2.495, *P* = 0.047 in early stage; HR = 2.929, *P* = 0.019 in advanced stage) and liver cancer (HR = 3.086, *P* < 0.001 in early stage; HR = 2.219, *P* = 0.049 in advanced stage). DM negatively influenced OS when the BMI was within the normal range in overall cancer (HR = 2.468, *P* < 0.001), lung cancer (HR = 2.297, *P* < 0.001), digestive tract cancer (HR = 2.354, *P* < 0.001), liver cancer (HR = 2.406, *P* = 0.001), leukemia (HR = 4.039, *P* < 0.001) and breast cancer (HR = 4.222, *P* = 0.008). Among those with BMI ≥ 24 kg/m^2^, DM played a role only in lung cancer (HR = 1.597, *P* = 0.037).

**Conclusions:**

Patients with diabetes tend to combine worse body composition and inflammation status, and that glycemic control can ameliorate the impairment of diabetes to some extent.

## Introduction

Cancer is currently one of the major diseases that threaten the health of residents (1), and 8–18% cancer patients have diabetes as a comorbid medical condition (2). According to the epidemiological survey conducted by the Chinese Medical Association, the total number of people with diabetes in mainland China is 129.8 million (3). China has become the country with the highest incidence of diabetes in the world (4). Diabetes were significantly related with higher cancer occurrence and mortality in many cancer types (5). Overall cancer risk was found significantly elevated with a standardized increased ratio of 1.15 (95% CI 1.12–1.19) and 1.25 (95% CI 1.21–1.30) in males and females, respectively (6). A systemic review of 23 studies demonstrated a 41% increased risk for long-term, all-cause mortality for diabetic patients compared with those without diabetes (7). Diabetes and associated metabolic disorders contribute directly or indirectly to cancer progression (8). Anaerobic glycolysis, known as Warburg, is classic in cancer. But Warburg does not mean glucose is unimportant. In contrast, hyperglycemia stimulates cancer proliferation (9). Most cancers predominantly express the glucose transporter 1, which has a high affinity for glucose. The increased glycolysis in cancer cells provides the materials necessary for nucleotide, amino acid, and lipid synthesis. Besides, advanced glycation end products (AGRs) due to hyperglycemia, and its receptors (RAGRs) have been reported to lead to oxidative stress and increased inflammation, which promotes cancer growth, angiogenesis, and metastases (10). Obesity, also a prevalence worldwide, is a risk factor of both diabetes and cancer (11, 12). Excessive adipose tissue generates oxidative stress by increased production of pro-inflammatory adipokines. And BMI is regarded as a typical measurement of obese. There have been numerous studies on the relationship between diabetes and cancer. However, at present, barely convinced evidence clearly explains the extent of the effect of diabetes in different cancer types, stages and body mass indices (BMI), which remains to be further investigated (13–15). In addition, as a wasting disease, the nutritional status of patients gradually deteriorates with the development of cancers (16). Therefore, this large-scale retrospective cohort study is to investigate the impact of diabetes on the prognosis of tumor patients.

## Data and Methods

### Clinical Data Collection

Clinical data of cancer patients from November 2011 to December 2018 in the Department of Oncology, Cancer Center, First Hospital of Jilin University were collected. No specific selection criteria were established for cancer type or demographic characteristics, except for patients who declined to participate in the study. All patients were regularly followed up by telephone interviews or outpatient visits.

#### Main Inclusion Criteria

(1) Clear diagnosis of malignancy in pathological specimens. (2) Age ≥18 years. (3) No nutritional support treatment prior to nutritional assessment and laboratory testing.

#### Major Exclusion Criteria

(1) Those who were unwilling to keep blood specimens. (2) Combination of other types of tumors. (3) Combination of other metabolic or immunological diseases. (4) Those who had incomplete records of necessary indexes. Clinical-pathological variables including age, sex, BMI, tumor types, TNM stages (AJCC 7th edition), alcohol consumption, smoking status. Scales including Karnofsky Performance Status (KPS), the Patient-Generated Subjective Global Assessment (PG-SGA) and the Nutritional Risk Screening-2002 score (NRS-2002), and quality of life (QoL-C30). Laboratory examinations including total protein(TP), albumin, prealbumin (PAB), transferrin (TFN), C-reaction protein(CRP), neutrophil to lymphocyte ratio (NLR) and platelets to lymphocyte ratio (PLR). Anthropometric indices including hand-grip strength (HGS) and visceral fat area (VFA) by bioelectrical impedance analysis.

### Diabetes Diagnosis Criteria

According to the Diabetes Diagnosis and Treatment Criteria established by the American Diabetes Association (ADA), (1) Fasting blood glucose ≥7.0 mmol/L (overnight blood glucose without food for at least 8–10 h); (2) Oral glucose tolerance test (OGTT) two-hour blood glucose ≥11.1 mmol/L; (3) Hemoglobin A1c (HbA1c) ≥6.5%; (4) Random blood glucose ≥11.1 mmol/L, along with symptoms related to diabetes such as polydipsia, polyphagia, polyuria and emaciation. Meeting any one of the above four conditions can be diagnosed with diabetes mellitus.

### Classification of BMI

The BMI ranges were reclassified into normal (18.5 kg/m^2^-23.9 kg/m^2^), overweight (24.0 kg/m^2^-27.9 kg/m^2^) and obese (≥28 kg/m^2^) according to the BMI standards for Chinese adults published by the Working Group on Obesity (WGO).

### Operation Rules of Anthropometric Indices

HGS was examined in all subjects using a Jamar hydraulic grip dynamometer (Sammons Preston Rolyan, Illinois, USA). Patients were comfortably seated in an upright position with the shoulders tucked in, neutral rotation, 90°elbow flexion, and the forearms and wrists in a neutral position. The patient gripped the dynamometer with maximum strength. The test is performed three times in a row, with a 1-min rest at the end of each set, and the maximum grip strength is recorded.

VFA is assessed by the Inbody S10 (Biospace Co.^®^), a multi-frequency bioelectrical impedance body composition analyzer. For analysis on patients in the supine position, electrode pads are attached to the ipsilateral upper and lower extremities and all procedures are performed according to the manufacturer's recommendations.

### Statistical Analysis

Data were analyzed by SPSS for Windows version 26.0 (IBM SPSS Statistics, IBM Corp., Armonk, NY) and R version 4.0 (R Foundation for Statistical Computing, Vienna, Austria). All involved patients were classified into the non-DM group and DM group according to the ADA diagnosis criteria. The Kolmogorov–Smirnov test was used to confirm normal distributions of continuous data. Independent *t*-tests were used for normally distributed data. Counting data were examined by using the chi-square test. The Kaplan–Meier curve, log-rank test, and Cox regression analyses were used to perform survival analysis in specific cancer types, stages and BMI and in all participants. *P* < 0.05 was taken to indicate statistical significance.

## Results

Among the 2,929 cancer patients recruited, 43.4% were men and 56.6% were women, with a mean age of 55 years. The mean follow-up period was 36.24 months and 791 patients died. According to the ADA criteria for the management of DM, 2,533 patients were included in the non-DM group and 396 patients were included in the DM group. The demographic, clinical and pathological characteristics of patients in the non-DM and DM groups were shown in Table 1. DM was significantly associated with age, PG-SGA, VFA, tumor types and stages (P < 0.05).

**Table 1:** Patient characteristics stratified by diabetes.

**Characteristics**	**Groups**			
	**Non-DM (*n*%)**	**DM (*n*%)**	**Total**	***P*-value**
Age (year)				
<65	2,038 (80.5)	287 (72.5)	2,325 (79.4)	<0.001
≥65	495 (19.5)	109 (27.5)	604 (20.6)	
Sex				
Male	1,102 (43.5)	169 (42.7)	1,271 (43.4)	0.757
Female	1,431 (56.5)	227 (57.3)	1,658 (56.6)	
Smoking				
No	1,519 (60.0)	249 (62.9)	1,768 (60.4)	0.271
Yes	1,014 (40.0)	147 (37.1)	1,161 (39.6)	
Alcohol consumption				
No	2,050 (80.9)	327 (82.6)	2,377 (81.2)	0.437
Yes	483 (19.1)	69 (17.4)	552 (18.8)	
Tumor types				
Lung	773 (30.5)	125 (31.6)	898 (30.7)	0.001
Digestive tract	507 (20.0)	91(23.0)	598 (20.4)	
Liver	136 (5.4)	41 (10.4)	177 (6.0)	
Leukemia	284 (11.2)	31 (7.8)	315 (10.8)	
Breast	625 (24.7)	76 (19.2)	701(23.9)	
Others	208 (8.2)	32 (8.1)	240(8.2)	
TNM stages				
I	448 (18.6)	51 (13.3)	499 (17.9)	<0.001
II	569 (23.7)	86 (22.5)	655 (23.5)	
III	594 (24.7)	131 (34.2)	725 (26.0)	
IV	475 (19.8)	85 (22.2)	560 (20.1)	
Leukemia	318 (13.2)	30 (7.8)	348 (12.5)	
PG-SGA				
0–1	1,035 (40.9)	133 (33.6)	1,168 (39.9)	0.002
2–3	437 (17.3)	62 (15.7)	499 (17.1)	
4–8	744 (29.4)	130 (32.8)	874 (29.9)	
≥9	314 (12.4)	71 (17.9)	385 (13.2)	
NRS-2002				
<3	2,077 (90.7)	328 (92.7)	24,05 (90.9)	0.224
≥3	214 (9.3)	26 (7.3)	240 (9.1)	
QoL-C30				
<60	795 (31.5)	140 (35.4)	935 (32.0)	0.117
≥60	1,730 (68.5)	255 (64.6)	1,985 (68.0)	
VFA (cm^2^)				
<90	1,352 (53.4)	188 (47.5)	1,540 (52.6)	0.029
≥90	1,181 (46.6)	208 (52.5)	1,389 (47.4)	

*DM, diabetes mellitus; PG-SGA, the Patient-Generated Subjective Global Assessment; NRS-2002, the Nutritional Risk Screening-2002 score; QoL-C30, quality of life; VFA, visceral fat area*.

### Relationship Between DM and Clinical Parameters

Compared with non-DM patients, NRS-2002 was higher in DM patients with lung cancer (0.89 vs 0.64, *P* = 0.027) and breast cancer (0.42 vs 0.21, *P* = 0.034). BMI (23.92kg/m^2^ vs 23.15kg/m^2^, *P* = 0.016), VFA (99.31 vs 90.49cm^2^, *P* = 0.009), and NLR (5.88 vs 3.23, *P* = 0.003) were higher in patients with lung cancer combined with diabetes than in patients without diabetes. Digestive tract cancer patients with DM had higher BMI (23.24 vs 22.15kg/m^2^, *P* = 0.007), PLR (216.81 vs 180.08, *P* = 0.050) and VFA (91.94 vs 79.57cm^2^, *P* = 0.003) than non-DM patients. Liver cancer patients with DM had lower HGS compared with non-DM patients (22.85 vs 27.04kg, *P* = 0.031). TP (64.17 vs 59.36g/L, P < 0.001) and albumin (37.81 vs 35.42 g/L, *P* = 0.014) were higher, while VFA was lower (78.07 vs 95.98 cm^2^, *P* = 0.004) in patients with leukemia combined without diabetes than in patients with diabetes. In addition, HGS was lower (18.29 vs 20.38kg, *P* = 0.004) while NLR (5.98 vs 2.22, *P* < 0.001) and PLR (168.90 vs 146.04, *P* = 0.022) were higher in patients with breast cancer combined with diabetes than in patients without diabetes (Table 2).

**Table 2:** Basic clinical information for all involved patients stratified by DM.

**Parameters**	**Lung Cancer**			**Digestive tract Cancer**			**Liver Cancer**			**Leukemia**			**Breast Cancer**			**Others**		
	**(*****N*** **=** **898)**			**(*****N*** **=** **598)**			**(*****N*** **=** **177)**			**(*****N*** **=** **315)**			**(*****N*** **=** **701)**			**(*****N*** **=** **240)**		
	**Non-DM**	**DM**	***P***	**Non-DM**	**DM**	***P***	**Non-DM**	**DM**	***P***	**Non-DM**	**DM**	***P***	**Non-DM**	**DM**	***P***	**Non-DM**	**DM**	***P***
NRS2002	0.64	0.89	0.027	0.85	0.64	0.106	0.67	0.62	0.813	0.29	0.38	0.603	0.21	0.42	0.034	0.74	0.59	0.564
KPS	89.43	88.88	0.554	86.49	87.69	0.425	88.82	86.83	0.347	89.79	86.77	0.251	91.95	92.24	0.780	89.09	88.75	0.874
TP (g/L)	67.78	67.99	0.724	64.02	66.09	0.032	65.38	64.90	0.716	64.17	59.36	<0.001	69.12	70.36	0.069	68.11	69.56	0.282
Albumin (g/L)	39.26	38.62	0.151	37.15	37.41	0.664	36.90	35.20	0.084	37.81	35.42	0.014	41.73	41.76	0.961	39.50	38.60	0.370
PAB (g/L)	0.21	0.21	0.257	0.19	0.19	0.794	0.18	0.17	0.971	0.22	0.21	0.765	0.23	0.24	0.562	0.21	0.19	0.082
TFN (g/L)	4.18	2.20	0.669	2.41	2.48	0.696	2.33	2.29	0.801	10.21	3.33	0.728	2.72	2.47	0.412	2.37	2.32	0.786
CRP (mg/L)	15.12	22.39	0.072	24.37	20.46	0.431	12.18	18.23	0.375	26.70	22.52	0.614	5.80	9.57	0.340	26.43	16.22	0.494
Height (cm)	1.65	1.65	0.211	1.67	1.65	0.068	1.66	1.65	0.349	166.60	167.65	0.505	158.83	157.68	0.073	1.61	1.61	0.696
Weight (kg)	63.91	65.33	0.204	61.82	63.60	0.181	63.52	61.29	0.239	64.87	67.72	0.214	62.87	62.49	0.749	60.83	62.19	0.480
BMI (kg/m^2^)	23.15	23.92	0.016	22.15	23.24	0.007	22.89	22.36	0.328	23.30	24.07	0.258	24.90	25.10	0.641	23.43	23.84	0.513
HGS (kg)	26.34	25.11	0.197	25.94	23.89	0.068	27.04	22.85	0.031	25.61	26.69	0.580	20.38	18.29	0.004	21.15	20.40	0.616
VFA (cm^2^)	90.49	99.31	0.009	79.57	91.94	0.003	85.69	85.83	0.981	78.07	95.98	0.004	100.26	106.25	0.148	91.79	89.74	0.758
NLR	3.23	5.88	0.003	4.38	6.80	0.089	4.15	4.55	0.686	2.89	2.82	0.934	2.22	5.98	<0.001	3.87	3.01	0.059
PLR	171.55	174.12	0.839	180.08	216.81	0.050	147.52	185.18	0.074	174.68	184.85	0.795	146.04	168.90	0.022	188.54	173.66	0.611

*DM, diabetes mellitus; NRS-2002, the nutritional risk screening-2002 score; KPS, karnofsky performance status; TP, total protein; PAB, prealbumin; TFN, transferrin; CRP, C-reaction protein; BMI, body mass index; NLR, neutrophil to lymphocyte ratio; PLR, platelets to lymphocyte ratio; HGS, hand-grip strength; VFA, visceral fat area*.

### Relationship Between Glycemic Control and Clinical Indicators in DM Patients

Compared with non-DM patients, NRS-2002 was higher in E-DM patients with lung cancer (1.07 vs 0.64, *P* = 0.004) and breast cancer (0.64 vs 0.21, *P* = 0.001). DM patients with euglycemia (E-DM) had higher NLR than non-DM patients in lung cancer (4.31 vs 3.23, *P* = 0.014), gastrointestinal tumors (9.06 vs 4.34, *P* = 0.017), and breast cancer (3.40 vs 2.22, *P* = 0.014) (Table 3). Although the differences were not always statistically significant, E-DM patients had lower TFN, lower albumin and lower HGS than non-DM patients. It indicated that good glycemic control can make up the adverse effects of diabetes to some extent.

**Table 3:** Basic clinical information of E-DM vs. non-DM patients.

**Parameters**	**Lung Cancer**			**Digestive tract Cancer**			**Liver Cancer**			**Leukemia**			**Breast Cancer**			**Others**		
	**(*****N*** **=** **898)**			**(*****N*** **=** **598)**			**(*****N*** **=** **177)**			**(*****N*** **=** **315)**			**(*****N*** **=** **701)**			**(*****N*** **=** **240)**		
	**Non-DM**	**E-DM**	***P***	**Non-DM**	**E-DM**	***P***	**Non-DM**	**E-DM**	***P***	**Non-DM**	**E-DM**	***P***	**Non-DM**	**E-DM**	***P***	**Non-DM**	**E-DM**	***P***
NRS2002	0.64	1.07	0.004	0.85	0.71	0.513	0.67	0.92	0.478	0.29	0.80	0.069	0.21	0.64	0.001	0.75	0.57	0.540
KPS	89.41	88.85	0.636	86.63	86.88	0.901	88.82	85.00	0.254	89.79	87.14	0.341	91.97	91.72	0.877	89.01	88.70	0.899
TP (g/L)	67.78	66.99	0.345	64.16	66.18	0.072	65.38	64.29	0.607	64.17	56.00	<0.001	69.13	69.65	0.625	68.02	69.03	0.521
Albumin (g/L)	39.26	38.29	0.114	37.23	37.59	0.652	36.90	34.89	0.191	37.81	33.76	0.004	41.74	41.02	0.348	39.47	38.64	0.475
PAB (g/L)	0.21	0.21	0.529	0.19	0.18	0.465	0.18	0.15	0.270	0.22	0.18	0.127	0.23	0.22	0.251	0.21	0.18	0.092
TFN (g/L)	4.19	2.08	0.748	2.42	2.39	0.910	2.33	2.20	0.577	2.49	2.40	0.891	2.72	2.58	0.738	2.37	2.27	0.649
CRP (mg/L)	15.16	17.41	0.618	23.40	23.26	0.983	12.18	9.08	0.774	10.21	5.63	0.321	5.80	12.00	0.246	26.66	17.36	0.579
Height (cm)	1.66	1.64	0.098	1.67	1.66	0.453	1.66	1.63	0.097	1.67	1.69	0.250	1.59	1.57	0.140	1.61	1.61	0.949
Weight (kg)	63.81	62.69	0.457	61.79	63.39	0.362	63.52	60.01	0.240	64.87	64.48	0.907	62.87	60.42	0.185	60.77	61.03	0.907
BMI (kg/m^2^)	23.12	23.14	0.963	22.16	22.97	0.128	22.89	22.66	0.785	23.30	22.49	0.406	24.91	24.38	0.435	23.37	23.48	0.881
HGS (kg)	26.32	24.27	0.119	26.00	24.12	0.203	27.04	18.02	0.003	25.61	24.21	0.622	20.38	17.33	0.008	21.21	19.64	0.363
VFA (cm^2^)	90.22	94.22	0.384	79.45	84.93	0.319	85.69	83.10	0.788	78.07	79.29	0.891	100.28	99.47	0.901	91.25	88.44	0.710
NLR	3.23	4.31	0.014	4.34	9.06	0.017	4.15	4.50	0.822	2.89	2.73	0.892	2.22	3.40	<0.001	3.88	2.85	0.047
PLR	171.69	182.99	0.529	179.75	234.08	0.032	147.52	210.40	0.051	174.68	194.66	0.727	146.10	183.39	0.011	188.47	178.58	0.775

*DM, diabetes mellitus; E-DM, euglycemia diabetes mellitus; NRS-2002, the nutritional risk screening-2002 score; KPS, karnofsky performance status; TP, total protein; PAB, prealbumin; TFN, transferrin; CRP, C-reaction protein; BMI, body mass index; NLR, neutrophil to lymphocyte ratio; PLR, platelets to lymphocyte ratio; HGS, hand-grip strength; VFA: visceral fat area*.

### Prognostic Impact of DM in Different Cancer Types Stratified by Stages

**Figure 1:**
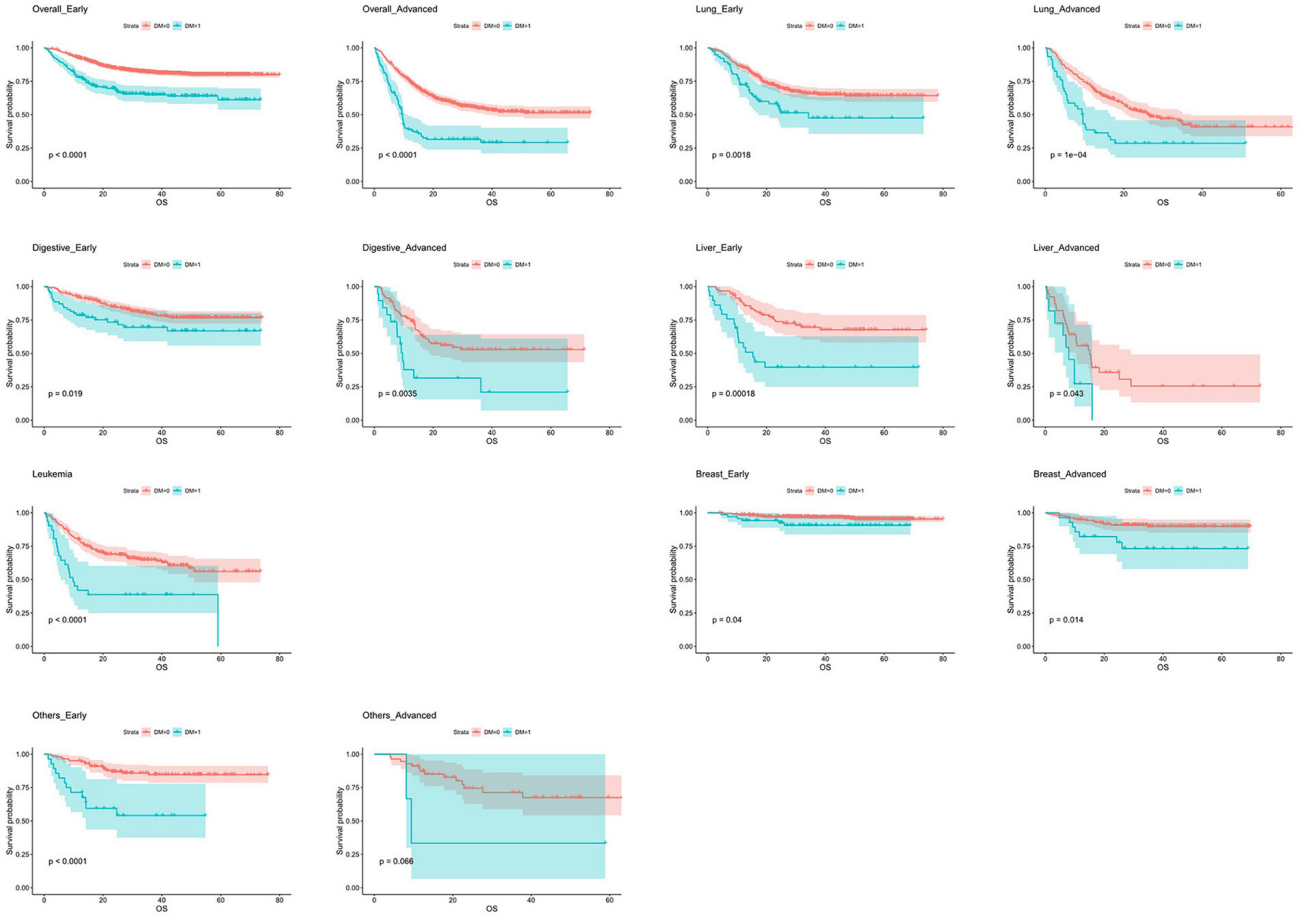
Kaplan–Meier curves of overall survival in different cancer types stratified by stage. DM, diabetes mellitus.

Table 4 and Figure 1 showed the relationship between DM and OS in specific cancer types stratified by stages. Compared with non-DM patients, OS in DM patients was significant shorter in overall cancer, lung cancer, gastrointestinal tract tumors, leukemia, advanced stage breast cancer and early stage liver cancer. The HR was 2.599 (95% CI: 2.024–3.336, *P* < 0.001) in early stage overall cancer, 2.427 (95% CI: 1.887–3.121, *P* < 0.001) in advanced overall cancer, 2.076 (95% CI: 1.332–3.236, *P* = 0.001) in early stage lung cancer, 2.118 (95% CI: 1.437–3.121, *P* < 0.001) in advanced lung cancer, 1.768 (95% CI: 1.093–2.863, *P* = 0.020), in early stage digestive tract cancer, 2.454 (95% CI: 1.316–4.576, *P* = 0.005) in advanced digestive tract cancer, 3.086 (95% CI: 1.668-5.708, *P* < 0.001) in early stage liver cancer, 2.219 (95% CI: 1.004-4.906, *P* = 0.049) in advanced liver cancer, 2.636 (95% CI: 1.628–4.269, *P* < 0.001) in leukemia, 2.495 (95% CI: 1.011–6.155, *P* = 0.047) in early stage breast cancer, 2.929 (95% CI: 1.193–7.189, *P* = 0.019) in advanced breast cancer, and 4.320 (95% CI:2.103–8.871, *P* < 0.001) in patients with early stage other tumors (Figure 1).

**Table 4:** Hazard risk for all cancers mortality in patients with diabetes stratified by stages.

**Specific tumor types**	**HR**	**95% CI**	***P*-values**
Overall cancer			
Early	2.599	2.024–3.336	<0.001
Advanced	2.427	1.887–3.121	<0.001
Lung cancer			
Early	2.076	1.332–3.236	0.001
Advanced	2.118	1.437–3.121	<0.001
Digestive tract cancer			
Early	1.768	1.093–2.863	0.020
Advanced	2.454	1.316–4.576	0.005
Liver cancer			
Early	3.086	1.668–5.708	<0.001
Advanced	2.219	1.004–4.906	0.049
Leukemia	2.636	1.628–4.269	<0.001
Breast Cancer			
Early	2.495	1.011–6.155	0.047
Advanced	2.929	1.193–7.189	0.019
Others			
Early	4.320	2.103–8.871	<0.001
Advanced	3.691	0.830–16.411	0.086

### Prognostic Impact of DM in Different Cancer Types Stratified by BMI

**Figure 2:**
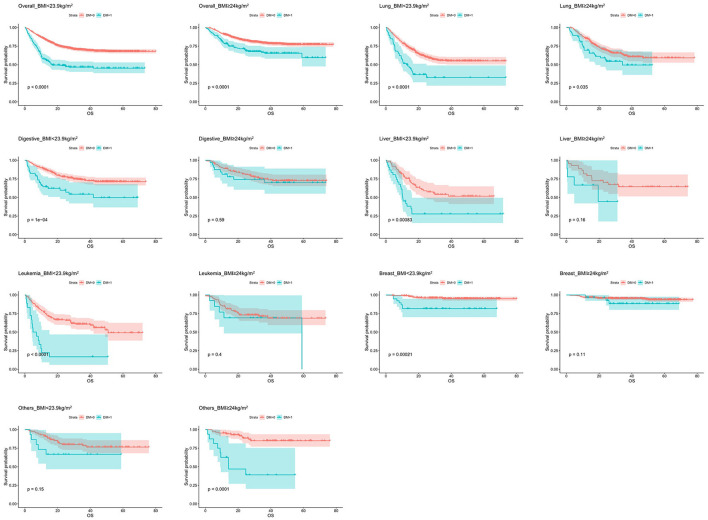
Kaplan–Meier curves of overall survival in different cancer types stratified by BMI.

Patients was classified by BMI into normal (18.5 ≤ BMI < 23.9 kg/m^2^) and obese (BMI ≥ 24.0 kg/m^2^) categories according to WGO. Table 5 and Figure 2 showed the relationship between DM and OS in specific cancer types stratified by BMI. The combination of diabetes had negative impact on OS when the BMI was within the normal range in lung cancer (HR = 2.297, 95% CI: 1.635–3.229, *P* < 0.001), digestive tract cancer (HR = 2.354, 95% CI: 1.508–3.675, *P* < 0.001), liver cancer (HR = 2.406, 95% CI: 1.414–4.092, *P* = 0.001), leukemia (HR = 4.039, 95% CI: 2.291–7.120, *P* < 0.001) and breast cancer (HR = 4.222, 95% CI: 1.466–12.164, *P* = 0.008). In contrast, among those with BMI ≥ 24 kg/m^2^, DM played a role only in lung cancer (HR = 1.597, 95% CI: 1.029–2.479, *P* = 0.037) and other tumors (HR = 6.747, 95% CI: 2.769–16.441, *P* < 0.001). Furthermore, it was observed that, the HR of DM patients with BMI in normal range (HR = 2.468, 95% CI: 2.004–3.041, *P* < 0.001) was significantly higher than those whose BMI ≥ 24 kg/m^2^ (HR = 1.898, 95% CI: 1.410–2.556, *P* < 0.001) in overall tumors.

**Table 5:** Hazard risk for all cancers mortality in patients with diabetes stratified by BMI (kg/m^2^).

**Specific tumor types**	**HR**	**95% CI**	***P*-values**
Overall Cancer			
18.5 ≤ BMI < 23.9	2.468	2.004 to 3.041	<0.001
BMI ≥ 24	1.898	1.410 to 2.556	<0.001
Lung Cancer			
18.5 ≤ BMI < 23.9	2.297	1.635 to 3.229	<0.001
BMI ≥ 24	1.597	1.029 to 2.479	0.037
Digestive tract Cancer			
18.5 ≤ BMI < 23.9	2.354	1.508 to 3.675	<0.001
BMI ≥ 24	1.220	0.588 to 2.534	0.594
Liver Cancer			
18.5 ≤ BMI < 23.9	2.406	1.414 to 4.092	0.001
BMI ≥ 24	2.203	0.718 to 6.762	0.168
Leukemia			
18.5 ≤ BMI < 23.9	4.039	2.291 to 7.120	<0.001
BMI ≥ 24	1.496	0.580 to 3.858	0.405
Breast Cancer			
18.5 ≤ BMI < 23.9	4.222	1.466 to 12.164	0.008
BMI ≥ 24	1.526	0.447 to 5.210	0.500
Others			
18.5 ≤ BMI < 23.9	2.002	0.760 to 5.271	0.160
BMI ≥ 24	6.747	2.769 to 16.441	<0.001

## Discussion

In previous studies, significant differences in inflammatory status, nutritional status, and quality of life between diabetic and non-diabetic patients have been demonstrated (17, 18). But there was no large-scale data on the differences between diabetic and non-diabetic patients and whether effective glycemic control can make up the adverse effects of diabetes in cancer patients. In the present study, we found that body composition and inflammatory parameters in cancer patients with diabetes differed from those without diabetes, suggesting that diabetes exacerbated the systemic inflammatory response of the body (19). For diabetic patients with good glycemic control, there still existed relatively more active inflammatory status compared to patients without diabetes, especially NLR. But quite a few indicators, such as albumin, PAB and CRP were not statistically different, suggesting that good glycemic control can reduce the adverse effects of diabetes to some extent.

DM has a negative impact on tumor patients in different stages. For most types of tumors, the prognosis of patients with DM is poor (20). Diabetes has a greater impact on patient prognosis in early stage liver cancer patients due to the combination of systemic metabolic changes and the development of systemic inflammation in patients with liver cancer at early stages (21). However, DM behaved as a stronger risk factor in advanced patients compared with those in early stage in colon cancer, lung cancer and breast cancer, which may due to the longer survival period of advanced cancer that allows the risk of DM to unfold. Therefore, the management of blood glucose in cancer patients with relatively longer survivals becomes increasingly important (22). It is worth mentioning that the adverse effects of diabetes were observed in leukemia, which may further illustrate the role of abnormal metabolism in malignant hematologic diseases and provide a theoretical basis for subsequent studies on the mechanisms of hematologic metabolism.

The BMI stratification was also discussed, and the risk of diabetes was more pronounced in patients with normal BMI. Patients with normal BMI and diabetes tend to have a longer disease duration and are less tolerant of treatment, while patients with higher BMI even without diabetes continuously exist a similar systemic inflammatory response as diabetic patients (23), which may account for the differences in diabetes risk across populations with different BMIs.

There is a limitation for choosing blood glucose as a marker of glycemic control mainly for the shortcomings of possibly inaccurate assessment of long term blood glucose control. The reasons are listed following. First, the glycated hemoglobin A1C (HbA1c), a reliable measurement of glycemic control, was not chose in this study as a marker of glycemic control mainly due to the inconvenience. In real clinical settings, HbA1c was mainly tested when DM was newly diagnosed or a DM patient with unsatisfied blood glucose levels. Second, although the cutoff value of HbA1c for DM is clearly defined, the reasonable threshold of HbA1c for predicting the prognosis was not a consensus (24), which may bring bias if HbA1c was chose as a glycemic control marker. Third, measurements of DM which also have an impact on cancer development should be taken into analysis as confounders. Several diabetes medications are reported related with cancer prognosis, typically metformin. To be accurate, the mechanisms are still unclear and the results of metformin in cancer prognosis are not always positive (25, 26). Given the limited patients using metformin (only 1.2%) in this study, the stratified sub-analysis was not committed. This topic should be further investigated.

In summary, we found that patients with diabetes tend to combine worse body composition and inflammatory indicators, and that glycemic control can ameliorate the impairment of diabetes to some extent. The above results suggest the negative influence of hyperglycemia in systemic inflammation metabolism. Besides, the risk posed by diabetes is not the same in patients with different tumor types and stages. Thus, the management of diabetes should be emphasized, especially for patients in early stages, which may bring a more durable disease-free state. Finally, we analyzed patients with different BMI to further analyze the relationship between body composition and diseases, and we believe that more studies should be done in the future.

## Data Availability Statement

The original contributions presented in the study are included in the article/supplementary material, further inquiries can be directed to the corresponding authors.

## Author Contributions

XL, KZ, and WJ conducted and drafted the manuscript. WZ and YL collected and analyzed the data. ML, JC, and WL designed the manuscript. ML, JC, WL, XL, KZ, and WJ revised the manuscript. All authors listed have made a substantial, direct, and intellectual contribution to the work and approved it for publication.

## Funding

This research was supported by Jilin Province Health Technology Innovation Project (No. 2017J064).

## Conflict of Interest

The authors declare that the research was conducted in the absence of any commercial or financial relationships that could be construed as a potential conflict of interest.

## Publisher's Note

All claims expressed in this article are solely those of the authors and do not necessarily represent those of their affiliated organizations, or those of the publisher, the editors and the reviewers. Any product that may be evaluated in this article, or claim that may be made by its manufacturer, is not guaranteed or endorsed by the publisher.

## References

[1] Risch HA. Diabetes and pancreatic cancer: both cause and effect. J Natl Cancer Inst. (2019) 111:1–2. 10.1093/jnci/djy093 29917095

[2] Shahid RK Ahmed S Le D Yadav S. Diabetes and cancer: risk, challenges, management and outcomes. Cancers (Basel). (2021) 13:5735. 10.3390/cancers13225735 34830886 PMC8616213

[3] Lega IC Lipscombe LL. Review: diabetes, obesity, and cancer-pathophysiology and clinical implications. Endocr Rev. (2020) 41:bnz014. 10.1210/endrev/bnz014 31722374

[4] Fang Y Zhang X Xu H Smith-Warner SA Xu D Fang H. Cancer risk in Chinese diabetes patients: a Retrospective cohort study based on management data. Endocr Connect. (2018) 7:1415–23. 10.1530/EC-18-0381 30475218 PMC6300864

[5] Ren X Zhang X Zhang X Gu W Chen K Le Y. Type 2 diabetes mellitus associated with increased risk for colorectal cancer: evidence from an international ecological study and population-based risk analysis in China. Public Health. (2009) 123:540–4. 10.1016/j.puhe.2009.06.019 19664792

[6] Wang M Hu RY Wu HB Pan J Gong WW Guo LH. Cancer risk among patients with type 2 diabetes mellitus: a population-based prospective study in China. Sci Rep. (2015) 5:11503. 10.1038/srep11503 26082067 PMC4469976

[7] Barone BB Yeh HC Snyder CF Peairs KS Stein KB Derr RL. Long-term all-cause mortality in cancer patients with preexisting diabetes mellitus: a systematic review and meta-analysis. JAMA. (2008) 300:2754–64. 10.1001/jama.2008.824 19088353 PMC3093051

[8] Shlomai G Neel B LeRoith D Gallagher EJ. Type 2 diabetes mellitus and cancer: the role of pharmacotherapy. J Clin Oncol. (2016) 34:4261–9. 10.1200/JCO.2016.67.4044 27903154 PMC5455318

[9] Vander Heiden MG Cantley LC Thompson CB. Understanding the Warburg effect: the metabolic requirements of cell proliferation. Science. (2009) 324:1029–33. 10.1126/science.1160809 19460998 PMC2849637

[10] Rojas A González I Morales E Pérez-Castro R Romero J Figueroa H. Diabetes and cancer: Looking at the multiligand/RAGE axis. World J Diabetes. (2011) 2:108–13. 10.4239/wjd.v2.i7.108 21860695 PMC3158864

[11] Gallagher EJ LeRoith D. Obesity and Diabetes: The Increased Risk of Cancer and Cancer-Related Mortality. Physiol Rev. (2015) 95:727–48. 10.1152/physrev.00030.2014 26084689 PMC4491542

[12] Kashfi K Rosen CL Aslan M. Obesity, type-2 diabetes and cancer: mechanistic insights. Crit Rev Oncog. (2019) 24:285–305. 10.1615/CritRevOncog.2019032959 32422026 PMC11034808

[13] Xu Y Wang L He J Bi Y Li M Wang T. Prevalence and control of diabetes in Chinese adults. JAMA. (2013) 310:948–59. 10.1001/jama.2013.168118 24002281

[14] Wang L Gao P Zhang M Huang Z Zhang D Deng Q. Prevalence and ethnic pattern of diabetes and prediabetes in China in 2013. JAMA. (2017) 317:2515–23. 10.1001/jama.2017.7596 28655017 PMC5815077

[15] Li Y Teng D Shi X Qin G Qin Y Quan H. Prevalence of diabetes recorded in mainland China using 2018 diagnostic criteria from the American Diabetes Association: national cross sectional study. BMJ. (2020) 369:m997. 10.1136/bmj.m997 32345662 PMC7186854

[16] Mayne ST Playdon MC Rock CL. Diet, nutrition, and cancer: past, present and future. Nat Rev Clin Oncol. (2016) 13:504–15. 10.1038/nrclinonc.2016.24 26951041

[17] Benhamou PY Somers F Lablanche S Debaty I Borel AL Nasse L. Impact of flexible insulin therapy on blood glucose variability, oxidative stress and inflammation in type 1 diabetic patients: the VARIAFIT study. Diabetes Metab. (2014) 40:278–83. 10.1016/j.diabet.2014.01.004 24581956

[18] Lontchi-Yimagou E Sobngwi E Matsha TE Kengne AP. Diabetes mellitus and inflammation. Curr Diab Rep. (2013) 13:435–44. 10.1007/s11892-013-0375-y 23494755

[19] Cignarelli A Genchi VA Caruso I Natalicchio A Perrini S Laviola L. Diabetes and cancer: Pathophysiological fundamentals of a 'dangerous affair'. Diabetes Res Clin Pract. (2018) 143:378–88. 10.1016/j.diabres.2018.04.002 29679627

[20] Srivastava SP Goodwin JE. Cancer biology and prevention in diabetes. Cells. (2020) 9:1380. 10.3390/cells9061380 32498358 PMC7349292

[21] Pang Y Kartsonaki C Turnbull I Guo Y Clarke R Chen Y. Diabetes, plasma glucose, and incidence of fatty liver, cirrhosis, and liver cancer: a prospective study of 0.5 million people hepatology. (2018) 68:1308–18. 10.1002/hep.30083 29734463 PMC6220764

[22] García-Jiménez C Gutiérrez-Salmerón M Chocarro-Calvo A García-Martinez JM Castaño A. De la Vieja A. From obesity to diabetes and cancer: epidemiological links and role of therapies. Br J Cancer. (2016) 114:716–22. 10.1038/bjc.2016.37 26908326 PMC4984860

[23] Kim GR Choi DW Nam CM Jang SI Park EC. Synergistic association of high-sensitivity C-reactive protein and body mass index with insulin resistance in non-diabetic adults. Sci Rep. (2020) 10:18417. 10.1038/s41598-020-75390-1 33116232 PMC7595183

[24] Qian J Wang W Wang L Lu J Zhang L Zhang B. The survival benefit for optimal glycemic control in advanced non-small cell lung cancer patients with preexisting diabetes mellitus. Front Oncol. (2021) 11:745150. 10.3389/fonc.2021.745150 34868942 PMC8635102

[25] Skinner H Hu C Tsakiridis T Santana-Davila R Lu B Erasmus JJ. Addition of metformin to concurrent chemoradiation in patients with locally advanced non-small cell lung cancer: The NRG-LU001 phase 2 randomized clinical trial. JAMA Oncol. (2021) 7:1324–32. 10.1001/jamaoncol.2021.2318 34323922 PMC8323052

[26] Tsakiridis T Pond GR Wright J Ellis PM Ahmed N Abdulkarim B. Metformin in combination with chemoradiotherapy in locally advanced non-small cell lung cancer: The OCOG-ALMERA randomized clinical trial. JAMA Oncol. (2021) 7:1333–41. 10.1001/jamaoncol.2021.2328 34323924 PMC8323053

